# European cephalopods distribution under climate-change scenarios

**DOI:** 10.1038/s41598-021-83457-w

**Published:** 2021-02-16

**Authors:** Alexandre Schickele, Patrice Francour, Virginie Raybaud

**Affiliations:** Université Côte d’Azur, CNRS, UMR 7035 ECOSEAS, Nice, France

**Keywords:** Biogeography, Ecological modelling, Marine biology, Climate-change impacts, Projection and prediction

## Abstract

In a context of increasing anthropogenic pressure, projecting species potential distributional shifts is of major importance for the sustainable exploitation of marine species. Despite their major economical (i.e. important fisheries) and ecological (i.e. central position in food-webs) importance, cephalopods literature rarely addresses an explicit understanding of their current distribution and the potential effect that climate change may induce in the following decades. In this study, we focus on three largely harvested and common cephalopod species in Europe: *Octopus vulgaris*, *Sepia officinalis* and *Loligo vulgaris*. Using a recently improved species ensemble modelling framework coupled with five atmosphere–ocean general circulation models, we modelled their contemporary and potential future distributional range over the twenty-first century. Independently of global warming scenarios, we observed a decreasing in the suitability of environmental conditions in the Mediterranean Sea and the Bay of Biscay. Conversely, we projected a rapidly increasing environmental suitability in the North, Norwegian and Baltic Seas for all species. This study is a first broad scale assessment and identification of the geographical areas, fisheries and ecosystems impacted by climate-induced changes in cephalopods distributional range.

## Introduction

Cephalopods represent a major and increasingly targeted group by fisheries worldwide with annual landings ranging from 2 million tons in 1980 to 4 million tons in 2010 (c.a. 2–4% of global annual landings), respectively generating 3 to 8 billion US$ per year (c.a. 2–5% of global landing value)^1^. They are intermediate trophic level and opportunistic species that occupy a central role in the food webs of temperate ecosystems^2–4^. They feed mainly on benthic and demersal communities (e.g. fish, crustacean, Mollusca) and are mostly predated by marine mammals (e.g. seals, cetaceans) and piscivorous fishes (e.g. Sparidae, Serranidae)^3^. Most cephalopods are short lifespan species (c.a. 2–4 years) characterised by a rapid growth (i.e. maturity after one winter) and an important sensitivity to environmental conditions^5–7^. Environmental stress related to temperature (e.g. heatwaves) or salinity (e.g. important river discharge) may therefore affect the physiology (e.g. larval survival, growth, reproduction) of these small bodied and largely dispersing species (i.e. external fecundation, high number of gametes)^5,6,8,9^. By affecting these critical lifestages, environmental conditions are also defining the recruitment, abundance and distribution of cephalopod species^5^, which may influence their sustainable exploitation and economic importance^10^.

Since the mid-nineteenth-century, Earth has faced global and unprecedented anthropogenic changes, leading to an average temperature increase of 0.93 °C^11^. Temperature is currently increasing at a rate of + 0.2 °C per decade^12^, leading to a + 1.5 to + 4.5 °C average temperature increase by the end of the century, depending on global political, societal and demographical pathways^13,14^. Global climate change is therefore directly altering the living environment of marine species^15–17^, especially temperature (i.e. yearly average and extreme climatic events) that is of major importance in the lifecycle of cephalopods (e.g. size and number of eggs, growth rate)^5,10,18^. In this context, a global proliferation of cephalopods has been reported in recent years^19^, including locally observed distributional range shifts and an important climate-induced variability (e.g. spawning season, recruitment) in temperate seas (e.g. in the North and Yellow seas)^20–22^. According to these observed distributional and behavioural changes, recent predictions highlighted the potential capacity of cephalopods to extent their distribution towards the pole, suggesting a future range expansion of these species^23–25^. In addition, recent studies highlighted the major economic importance of fisheries for several European countries^26,27^. Medium to long-term distribution shift of cephalopods—that represent important capture in Europe^1,28^—may induce major costs and economic consequences related to an adaptation of their future exploitation strategies^29–31^. Anticipating these climate-induced changes is therefore necessary to avoid abrupt fisheries adaptations at higher costs^29^. In a context of severe, climate-induced warming in European seas (up to + 0.35 °C per decade)^32,33^, a sustainable resource management perspective^34^ and the sensitivity of cephalopods to environmental variations^10,35^, it is therefore of major importance to project robust scenarios of cephalopod responses to changing environmental conditions in Europe.

These interactions between environmental conditions and species are formalised in the concept of ecological niche (sensu Hutchinson)^36,37^, that is defined as the *n*-dimensional ensemble of environmental conditions necessary for a species to live and reproduce. Based on this concept, Species Distribution Models define the potential distribution of a species according to the same *n*-dimensional ensemble of environmental conditions in which the species is observed^38,39^. Conversely to other modelling approaches (e.g. habitat or ecosystem models), SDMs are based on occurrence data, encompassing the entire distributional range of a species^38^. By considering the entire range of suitable environmental conditions, SDMs are able to estimate the global distributional range of a species under past, present and future climate conditions^39^. Ensemble models, that are SDMs constructed from several statistical algorithms using the same environmental factors, estimate an average species response to environmental conditions and its related uncertainty, avoiding a-priori assumptions on its shape^40–42^. Coupled with several climate models, these multi-algorithm procedures are known to produce a robust assessment of both contemporary and future distribution as well as the uncertainty associated to niche estimation and climate projections^40–43^.

In this study, we projected the contemporary and future potential distributions at the European scale, based on the outputs of an ensemble model for three cephalopod species common to our study area^3,7^: the common octopus (*Octopus vulgaris*; Cuvier, 1797), the common cuttlefish (*Sepia officinalis*; Linnaeus, 1758) and the common squid (*Loligo vulgaris*; Lamarck, 1798). The three considered species are the most representative in terms of official landings^1,28^. Moreover, they are broadly distributed in the European seas, overlapping with a large diversity of environmental conditions. For the three cephalopod species, we projected (i) their contemporary (1990–2017) distribution and (ii) a range of potential future distributional response to climate change over the twenty-first century, based on occurrence records and a recently developed ensemble modelling procedure^44,45^. Our framework integrates a multi-SDM approach (i.e. including the uncertainty between algorithms)^40^ coupled with three greenhouse gases emission scenarios (i.e. Representative Concentration Pathways, RCP)^13,14^ and five atmosphere–ocean General Circulation Models (GCMs) from the 5th phase of the Coupled Model Intercomparison Project, resulting in robust future cephalopod distribution projections^43^. These projections are needed for medium- to long-term conservation strategies and further operational studies, by identifying geographical areas where cephalopods may be subject to strong environmental impacts, at large spatial scale.

## Materials and methods

### Data collection

#### Cephalopod occurrence records

To avoid a truncated niche estimation and in line with SDM best practices^46,47^, it is necessary to consider the entire observed distributional range in SDM, independently of the study area, to avoid biases in future projections such as an overestimation of potential distributional range regression^46,47^. We acknowledge that the plasticity of cephalopod may induce local adaptations to environmental conditions^10,19^, of interest for regional and integrated studies. However, such adaptations are of negligible spatial range in our global distributional range estimation^48^. Therefore, we collected occurrence records for all studied species, at the global scale, from three available public databases encompassing the up-to-date known distribution of the three species. The database considered were: the Ocean Biogeographic Information System (OBIS, http://www.iobis.org/), the Global Biodiversity Information Facility (GBIF, https://www.gbif.org/) and SeaLifeBase (https://www.sealifebase.org/). To create the most up-to-date observation datasets, we completed the dataset with observations retrieved from peer-reviewed articles (see Supplementary Appendix 1). We then performed a data cleaning procedure on each cephalopod dataset to (i) remove unreliable occurrences (e.g. preserved specimen or taxonomic confusion)^49^, (ii) discard duplicated records and (iii) ensure their temporal and locational reliability (e.g. data on land, longitudinal and/or latitudinal inversion). The resulting up-to-date observation datasets included: 3380 (41 literature-based) occurrences for the common octopus, 4671 (including 17 literature-based) occurrences for the common cuttlefish and 1676 (90 literature-based) occurrences for the common squid. Conversely to quantitative data (e.g. a number of individuals, biomass or abundance) that highly depends on the sampling protocol, SDMs only requires georeferenced observations (i.e. occurrence points) to estimate the environmental conditions in which a species is observed^38^. Such qualitative data are produced by various sources, independently of the sampling protocol (e.g. gear, mesh size), allowing scientific survey data to be included in our datasets (e.g. MEDITS, ICES trawling surveys) as well as diving observations and georeferenced fisheries catch. Finally, because the studied cephalopod species are rarely observed below 300 m depth^3,7^, a precautionary bathymetry threshold (− 1000 m; due to important bathymetrical variation in the Mediterranean where the continental shelf is reduced) was applied to remove inconsistent occurrences, at the risk of removing some deep and uncommon observations. For the three considered species, occurrence data were aggregated on a 0.1° × 0.1° resolution spatial grid, corresponding to the resolution of environmental factors.

#### Environmental data

We then collected environmental factors (Table 1) to model the ecological niche (sensu Hutchinson)^36,37^ of each cephalopod species. Environmental factors values were first calculated on a yearly basis and then averaged on the 1990–2017 contemporary period. Among temperature related factors, the Sea Bottom Temperature (SBT) range was calculated as the difference between the SBT of the warmest month and the SBT of the coldest month within a year while the SBTvar was calculated as the inter-month SBT variance within a year. To project the evolution of future environmental factors for a range of radiative forcing^13,14^, GCMs consider a large variety of 3-dimensional environmental factors such as ocean circulation, water temperature, salinity, primary production, carbon cycle dynamics, atmospheric temperature and aerosol concentrations e.g. Refs.^50,51^. Without being exhaustive, GCMs projections may diverge for parametrisation reasons such as a spatial resolution ranging between 0.1° and 0.5°^52^ or their ability to model carbon or water cycle feedbacks (e.g. influencing ice cover)^33,53,54^. Because of their complexity and the absence of a ‘better performing’ GCM, the choice of a unique algorithm may greatly influence our future distributional range projections^43,55^. Therefore, following an ensemble modelling principle^41^ that accounts for uncertainty relative to future environmental factor projections^40,43^, we considered five commonly used GCMs retrieved from the 5th phase of the Coupled Model Intercomparison Project (CMIP5; Table 1). While further modelling steps require the same spatial resolution between environmental and occurrence data, the native resolution of environmental factors ranged between 0.1° and 0.5°. Therefore, to use them simultaneously in the modelling process, maps of environmental factors were linearly interpolated on both latitude and longitude to meet a 0.1° × 0.1° resolution spatial grid, ranging from 70° N to 70° S and 180° E to 180° W, corresponding to their common geographical domain.

**Table 1:** Description of the environmental factors considered in the ensemble models and of the corresponding references.

Name	Description	Contemporary (1990–2017)	Future (2006–2099)
SSS^a^	Sea surface salinity (‰)	Levitus’ climatology^56^ completed with ICES data (http://www.ices.dk/)	
SBT^b^	Mean annual sea bottom temperature (°C)	CORA: Coriolis Ocean database for ReAnalysis^57^	IPSL^50,52^, MPI^58,59^, CNRM^51^, HadGEM^60^ and GISS^61^ models
SBTrange^b^	Mean annual sea bottom temperature range (°C)		
SBTvar^b^	Mean monthly sea bottom temperature variance (°C)		

^a^Environmental factors kept constant in time.

^b^Temperature corresponding to the bottom vertical layer down to a maximum depth of 500 m.

### Description of the modelling framework

#### Modelling algorithms considered

The Environmental Suitability Index (ESI; index between 0 and 1, reflecting the suitability of environmental conditions necessary for a species to live and reproduce^37,38^) of the three cephalopod species was modelled using our recent multi-SDM framework described in Schickele et al.^45^, that considers critical issues in species distribution modelling such as sampling bias, pseudo-absence selection, model evaluation and uncertainty quantification. As a full description of the framework is available in Schickele et al.^45^, we only briefly recall here the main steps. Our framework is based on an ensemble modelling procedure including the Non-Parametric Probabilistic Ecological Niche (NPPEN) model^62,63^ and seven algorithms retrieved from Biomod2^64,65^: (i) Generalized Linear Model (GLM), (ii) Generalized Additive Model (GAM), (iii) Generalized Boosting Model (GBM), (iv) Artificial Neural Network (ANN), (v) Flexible Discriminant Analysis (FDA), (vi) Multiple Adaptive Regression Splines (MARS) and (vii) Random Forest (RF). This large range of algorithms integrates regression-based (i.e. GLM, GAM, MARS), machine learning (i.e. GBM, ANN, RF, FDA) and profile (i.e. NPPEN) methods.

#### Environmental variable pre-treatment

We constructed a parsimonious set of environmental factors to be tested in the SDMs. Because most algorithms are sensitive to multicollinearity among predictors^66^, we only considered the most important factor among each set of intercorrelated factor (Pearson’s r > 0.7). The relative importance of environmental factor to be tested in the models were assessed by sequentially randomising each environmental factor and calculating the resulting contemporary distribution (i.e. bootstrap procedure)^44^. According to this procedure, we considered the mean Sea Bottom Temperature (SBT, i.e. commonly admitted as the main factor shaping species distribution)^48,67^ for all species. In addition, and to refine our modelled distributional range, we tested SBT range or SBT var and Sea Surface Salinity (SSS) as supplementary environmental factors in the models. To avoid model over-parametrisation, we considered bathymetry^68^ and distance to coast^69^ as a-posteriori filters in a hierarchical filtering procedure^70^: the ESI value was only considered if the corresponding geographical cell was included within the distance to coast filter (i.e. if tested in the models) or the bathymetry filter for cells outside the distance to coast threshold. A unique bathymetry filter has been set at 300 m, which corresponds to the commonly observed depth range of the cephalopod species considered^3,7^. To be able to represent potentially suitable coastal cells characterised by an absence of coastal shelf (e.g. Mediterranean Sea), we tested a supplementary 50 km distance to coast filter. The 50 km value corresponds to 5 geographical cells from the coast, encompassing the commonly observed maximum distance from the coast outside the continental shelf. The ensemble of environmental factor combination that have been tested are shown in Supplementary Appendix 2.

#### Environmental filtration procedure

In order to alleviate the effect of spatially heterogenous sampling effort, that may induce biases in the environmental space used by the models, we proceeded to an environmental filtration^71^: for each species and combination of environmental factors (e.g. SBT × SBT range; see Supplementary Appendix 2) tested in the models, we considered only a single occurrence record among each group of observations characterised by the same environmental values (e.g. SBT of 15 °C, SBT range of 10 °C). The resulting dataset represents the ensemble of environmental values in which a species has been observed, with the same weight given to each observed condition, independently of the geographical sampling effort^71^. This filtration has been performed in an environmental domain of 0.5 °C resolution for SBT-related factors and 0.5 resolution for SSS.

#### Pseudo-absence selection

For each combination of environmental factors, we then selected the pseudo-absences, necessary for the calibration of all algorithms but NPPEN. Note that unlike real absences that may be found within suitable environmental conditions due to a variety of local factors (e.g. species interactions, food availability), pseudo-absences are a proxy of unsuitable environmental conditions used as algorithm input data. According to the latest SDM recommendations^45,72^, pseudo-absences were randomly selected outside the corresponding restricted convex hull in equal number of occurrences^73^. A restricted convex hull is defined as a convex hull ^74^ constructed by excluding outer quantiles (e.g. 2.5 and 97.5)^45^, therefore controlling the roughness of the ecological niche edge by overlapping presence and pseudo-absence on its edge. Because cephalopods are widely distributed^3,7^, the generated convex hull occupies most of the available range of environmental conditions on Earth, only leaving a few combinations to be selected for pseudo-absences. To avoid selecting multiple pseudo-absences characterised by identical environmental conditions (i.e. contradictory with the environmental filtration procedure), we tested three restricted convex hulls (i.e. excluding the 2.5–97.5, 5–95 and 10–90 outer quantile), therefore enlarging the environmental pseudo-absence selection range and avoiding a rough ecological niche edge. The combined environmental filtration and convexhull-based pseudo-absence selection procedure alleviates over-prediction and the effects of sampling biases or discontinuities on the modelled distribution, overall increasing the capacity of the model to reflect observed distribution^45,71,74,75^.

#### Ensemble model selection

Finally, we evaluated the adequacy of contemporary distributions with the occurrence records by the mean of the Continuous Boyce Index (CBI)^76^ which is the most performant statistical evaluation metric available for presence/pseudo-absence datasets (see discussion in Leroy et al.^77^. The CBI calculation was performed using a 10-time random cross-validation procedure (70% of the data was used to calibrate the model while the 30% remaining were used for model evaluation only). We estimated that 10 cross-validation repetitions provided a calibration dataset representative of the post pseudo-absence selection dataset (see details in Supplementary Appendix 3). A SDM was statistically validated for CBI values over 0.5^78^. In addition, we assessed the ecological quality of the corresponding response curves^79^, discarding spurious responses to environmental factors (e.g. bimodal response to temperature). Therefore, an ensemble model is defined by statistical algorithms, each considering the same explanatory variables and characterised by both the CBI and the corresponding response curves meeting the aforementioned criterion (Supplementary Appendix 2).

### Future projections

Future ESI projections were modelled using three RCP scenarios: (i) a peak and decline scenario (RCP2.6), (ii) an intermediate emission scenario (RCP4.5) and (iii) a “business as usual” scenario (RCP8.5). To highlight medium- to long-term tendencies and alleviate the effect of inter-annual stochasticity, uncertainty in GCM predictions^53^, future ESI were averaged for three different decades, respectively 2030–2039, 2050–2059 and 2090–2099 at the same 0.1° × 0.1° spatial resolution than contemporary environmental data. In order to compare future distributional range shifts between RCPs and time periods, future ESI projections were also given in the form of distributional centroids. For each species, period and RCP, we calculated the respective distributional centroid as the ESI-weighted barycentre of all geographical cells composing the corresponding potential distribution^80^. Environmental gradients (e.g. temperature) are differently oriented in the Mediterranean Sea (i.e. East–West) than along the Atlantic façade (i.e. South–North). Coastlines, that act as large-scale factors limiting the centroid evolution, are also differently oriented between the Mediterranean Sea and the Atlantic façade. To alleviate conflicting effects of these factors between the Mediterranean Sea and the Atlantic façade, that may induce bias in the centroid evolution, we considered both regions separately. As temperature related factors (Table 1) originate from two different datasets (i.e. observation-based data for the contemporary period and GCM-based data for future projections), we performed Taylor diagrams^81^ on their common time period (i.e. 2006–2017) to assess potential biases between the two datasets (Supplementary Appendix 4). To alleviate these biases, we corrected the value of each geographical cell of the GCM-based future dataset (i.e. each GCMs, RCPs and periods) by the difference relative to the corresponding cell of the observation-based contemporary dataset. This procedure, already applied by Cristofari et al.^82^ and Péron et al.^83^, resulted in a perfect correlation (Pearson’s r = 1), no standard deviation and no root mean square difference between the two data sources. The resulting corrected future environmental factors and the corresponding anomalies relative to present are given in Supplementary Appendix 5.

## Results

### Model selection

Our modelling procedure selected the best ensemble models (Table 2, details in Supplementary Appendix 2) to estimate the potential contemporary (1990–2017) distribution of three European cephalopod species and therefore the corresponding future projections. For all three species, mean SBT and annual SBT range were the environmental factors best explaining their observed contemporary distribution (see model selection in Supplementary Appendix 2). SSS was selected as a third factor for both common cuttlefish and common squid. The NPPEN model was selected in the ensemble model for all three species. The restricted convex hull excluding the 10th and 90th outer quantile resulted in overall higher CBI values and a smoother distribution edge for these widely spread species. Finally, all three cephalopod species showed CBI values above 0.85, indicating a high level of confidence in our ensemble model projections.

**Table 2:** Selected ensemble models for each cephalopod species.

Species	Common octopus *Octopus vulgaris*	Common cuttlefish *Sepia officinalis*	Common squid *Loligo vulgaris*
Environmental factor(s)	SBT and SBTrange	SBT, SBTrange and SSS	SBT, SBTrange and SSS
Statistical algorithm(s)	GLM, GAM, ANN and NPPEN	NPPEN	ANN and NPPEN
Restricted convex hull quantiles	10–90	10–90	10–90
Distance to coast threshold (km)	0	0	50
Bathymetry threshold (m)	− 300	− 300	− 300
CBI	0.89	0.85	0.89

*SBT* Sea bottom temperature, *SSS* sea surface salinity, *GLM* generalised linear models, *GAM* generalised additive models, *ANN* artificial neural network, *NPPEN* non-parametric probabilistic ecological niche model, *CBI* Continuous Boyce Index.

### Contemporary environmental suitability

**Figure 1:**
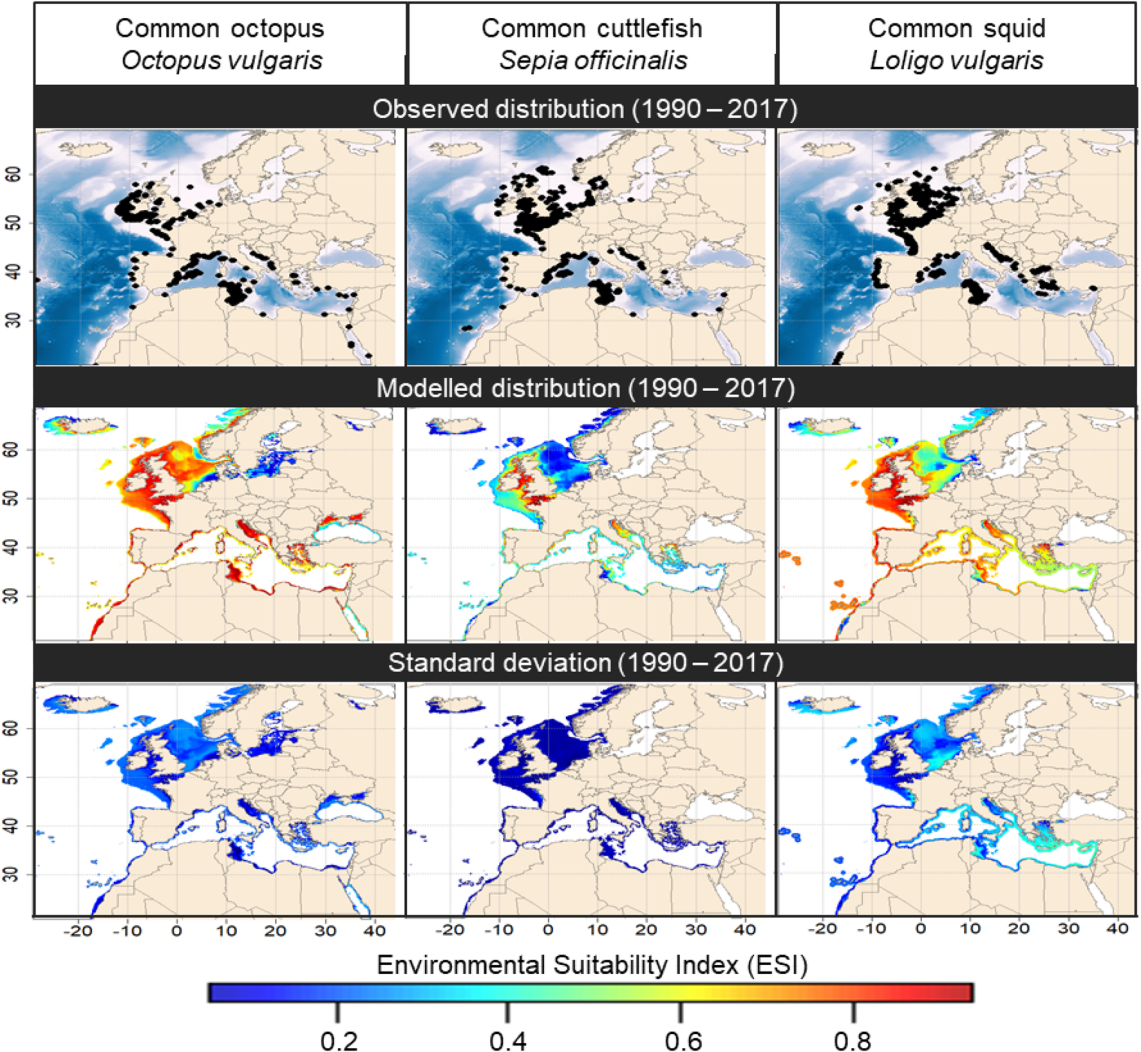
Top panels: observed contemporary (1990–2017) distribution in Europe of the three cephalopods species (*O. vulgaris, S. officinalis, L. vulgaris*). Each black dot represents an occurrence record. Middle panels: modelled contemporary (1990–2017) distribution represented in term of environmental suitability index ranging from 0 (low suitability) to 1 (maximum suitability). Bottom panels: corresponding standard deviation based on all SDM and cross-validation runs, of three studied cephalopod species in European waters. Note that a narrow distance to coast threshold has been added for common octopus and common cuttlefish for visual purposes only because of the coarse (i.e. 0.1°) coastal resolution. Maps were generated by A.S. using the R v3.4.4 software (R Core Team, 2018; https://www.R-project.org/), specifically the “raster” and “maptools” package. World borders were retrieved from http://thematicmapping.org.

Here we present the contemporary (1990–2017) distribution and the corresponding standard deviation (SD; i.e. including all SDM and cross-validation runs) of three studied cephalopod species in European waters (Fig. 1). Their global distributional range projections, corresponding to their calibration range necessary to avoid a truncated niche estimation, are available in Supplementary Appendix 6.

Based on the observed distribution (Fig. 1), all three cephalopod species are commonly found in the north-western Mediterranean Sea and along the European Atlantic façade. In accordance with the observed distribution, common octopus showed high ESI values (> 0.8) in the entire Mediterranean Sea and in the north-eastern Atlantic from Morocco to Norway. Moreover, we found moderate values of ESI in the Black Sea, the Norwegian coasts and the Baltic Sea to host suitable environmental conditions (ESI between 0.2 and 0.6) for this specie. We identified high ESI values (> 0.8) for the common cuttlefish along the western and southern British coasts and in a lesser extent in the northern Adriatic Sea. In addition, our models showed medium ESI values (between 0.4 and 0.6) for this specie along the Mediterranean Sea, the Portuguese coasts, the Bay of Biscay and the Celtic Sea. Finally, despite dense observations, the North Sea and the Gulf of Gabès present moderate-to-low ESI values (0.2–0.4) for this species. The common squid presents high ESI values (> 0.8) from the Celtic Sea and English Channel down to Morocco. We found medium to High ESI values (between 0.4 and 0.8) in the southern part of the North Sea and in the Mediterranean Sea, especially in northern Adriatic and Aegean Seas. Finally, we found the southern Norwegian coasts to host suitable environmental conditions for this species (ESI between 0.4 and 0.2).

The ensemble modelling framework includes an assessment of the modelling uncertainty (Fig. 1) between the different algorithms and cross-validation runs. In general, the SD is comprised between 0.1 and 0.3 depending on the geographical areas. We note that lower values are found for common cuttlefish because we only selected one statistical algorithm (i.e. NPPEN). The SD is spatially relatively homogeneous, indicating no major spatial bias in the modelling process. However, the SD is relatively high (0.4) for common squid in the south-eastern Mediterranean Sea compared to the associated ESI, indicating low confidence in the ESI values in this area and reflecting an abrupt niche slope. In such case, the presences and pseudo-absences variations between cross-validation runs as well as the variations between algorithms may induce important ESI variations.

### Future environmental suitability

**Figure 2:**
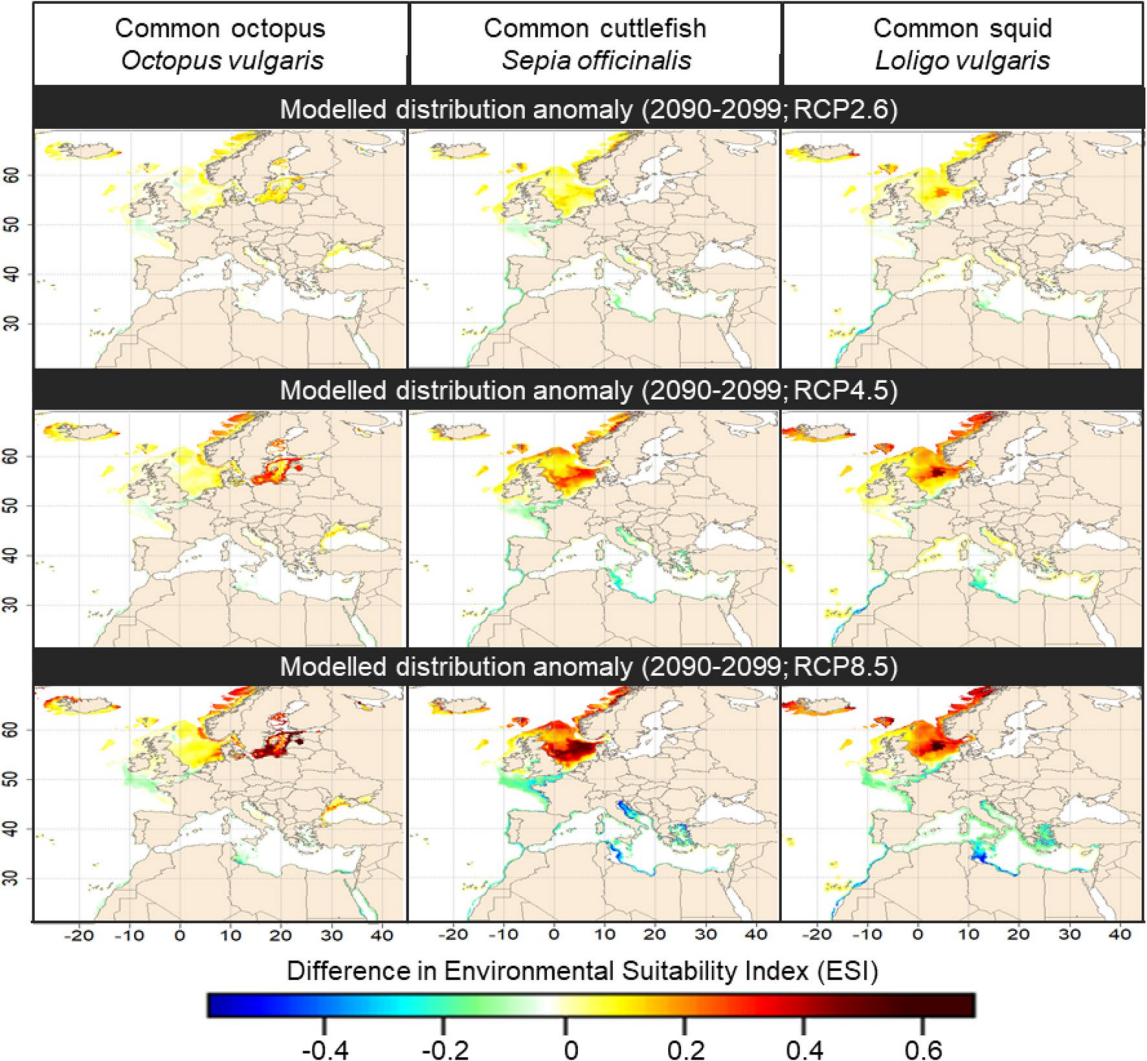
Future (2090–2099) environmental suitability anomalies (i.e. defined as the difference between future and contemporary ESIs) under RCP2.6, 4.5 and 8.5 conditions for Europe, relative to the contemporary period (1990–2017). Note that a narrow distance to coast threshold has been added for common octopus and common cuttlefish for visual purposes only because of the coarse (i.e. 0.1°) coastal resolution. Maps were generated by A.S. using the R v3.4.4 software (R Core Team, 2018; https://www.R-project.org/), specifically the “raster” and “maptools” package. World borders were retrieved from http://thematicmapping.org.

By coupling the potential environmental niche resulting from our ensemble models with RCP scenarios, we were able to project a range of potential future distribution for the three cephalopod species. Here we detail the late century projections under RCP2.6, 4.5 and 8.5 conditions (Fig. 2 and Supplementary Appendix 7).

For all three species, we expected a northward shift of the ESI along the European Atlantic façade (Fig. 2). Indeed, we projected ESI values to largely increase (up to + 0.6 under RCP8.5 conditions; Fig. 2) by the end of the century (2090–2099) in all areas located north of the English Channel. The highest ESI increases were expected in the central North Sea for common cuttlefish and common squid and in the Baltic sea for common octopus. On the contrary, we projected a general decrease in ESI values (down to − 0.4 under RCP8.5 conditions; Fig. 2) in the Bay of Biscay and the Mediterranean Sea for common cuttlefish, common squid and in a lesser extent for common octopus.

Resulting from these multiple changes, new associated ESI patterns are projected by the end of the century (Supplementary Appendix 7). The common octopus is expected to encounter high ESI values (> 0.8) along the entire European coasts except in the eastern part of the North Sea (ESI between 0.2 and 0.4) and in the Baltic Sea (ESI between 0.2 and 0.7). For the common cuttlefish, we projected areas characterised by high ESI values (> 0.8) to expand from the Celtic Sea toward the central North Sea under RCP4.5 conditions and up to the Norwegian coasts under RCP8.5 conditions (Supplementary Appendix 7). On the contrary, we expected low to medium ESI values (0.2–0.4) in the Mediterranean Sea with the highest values found in the Adriatic Sea and the Gulf of Lion. In addition, we projected environmental conditions outside the modelled environmental tolerance (ESI < 0.05) of this species in the southern Levantine Sea, that may strongly affect its future presence under RCP8.5 conditions. Finally, the European squid is projected to encounter high ESI values (> 0.8) along the Celtic Sea and the North Sea for all scenarios and in the Norwegian coasts under RCP8.5 conditions by the end of the century (Supplementary Appendix 7). However, low ESI values (from 0.2 to 0.4) are forecasted for this species along the eastern North Sea coastal area and the Bay of Biscay. In addition, we projected low to medium ESI values (between 0.2 and 0.6) in the Mediterranean Sea, with the highest values forecasted in the north-western Mediterranean basin.

For all species, we projected that the intensity of the projected distributional range shifts (Fig. 2 and Supplementary Appendix 7) is emphasised by severe warming (i.e. RCP8.5 conditions) and limited in case of a peak and decline scenario (RCP2.6).

### Future distributional centroid evolution

**Figure 3:**
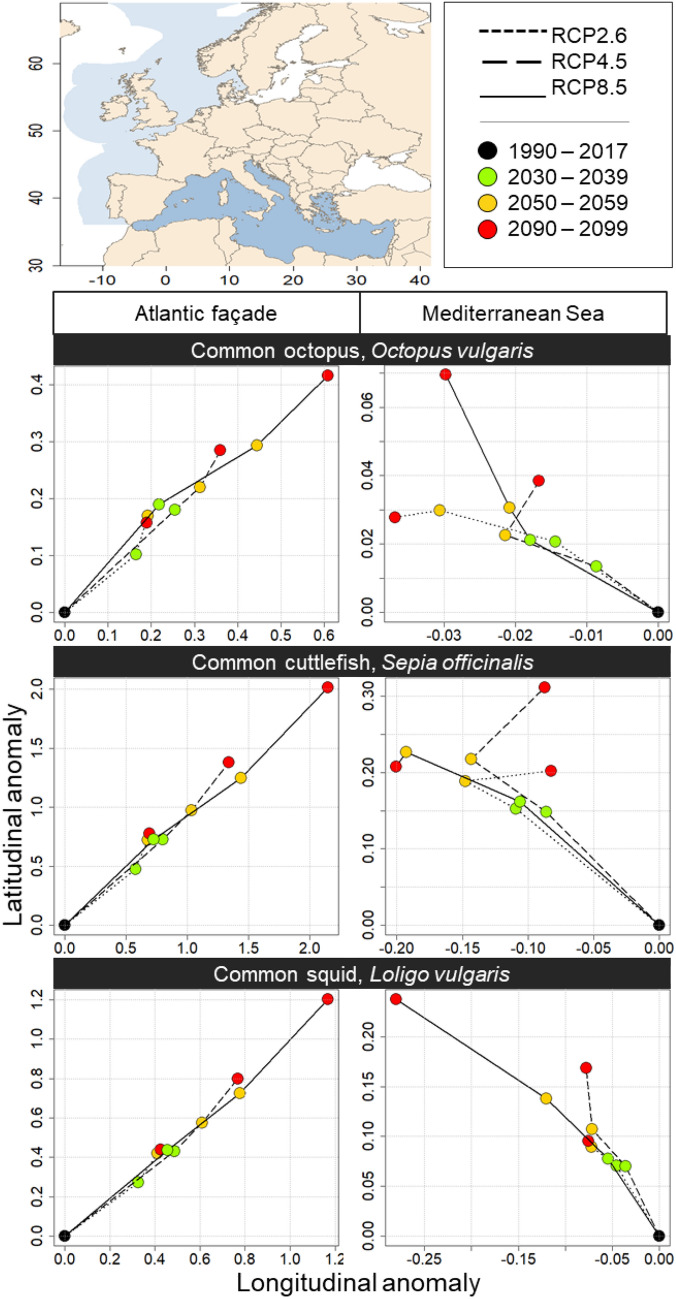
Distributional centroid evolution through space, time and climate change scenarios. The lines correspond to the climate change scenarios and the coloured dots to the different time periods. Left and right panels correspond to the geographical areas, respectively the European Atlantic façade and the Mediterranean Sea. Top, middle and bottom panels represent the three studied species, respectively common octopus, common cuttlefish, and common squid. Note the different scale in each plot. Maps were generated by A.S. using the R v3.4.4 software (R Core Team, 2018; https://www.R-project.org/), specifically the “sp” and “maptools” package. World borders were retrieved from http://thematicmapping.org.

The ensemble of potential future environmental suitability projections (i.e. 3 period and 3 RCPs) are synthesised through the evolution of the corresponding distributional centroid (Fig. 3). To minimise the effect of landmasses on the centroid evolution, the following results are separated between European Atlantic façade the and the Mediterranean Sea.

For all cephalopod species, we projected a north-eastward distributional centroid shift (up to + 2.0° N for common cuttlefish) along the European Atlantic façade (Fig. 3) in accordance with the projected ESI increase north of the English Channel (Fig. 2). The expected distributional centroid shift gradually increases over time (i.e. periods) and global warming intensity (i.e. RCPs). On a species level, it is more pronounced (e.g. for 2090–2099; RCP8.5) for common cuttlefish (up to + 2.0° N and + 2.0° E, Fig. 3) than for common squid (up to + 1.2° N and + 1.2° E, Fig. 3) and common octopus (up to + 0.4° N and + 0.6° E, Fig. 3). However, it is important to notice that the distributional centroid evolution is more important during the first half of the twenty-first century (i.e. from the contemporary period to the 2050–2059 decade) than during the second (i.e. from the 2050–2059 decade to the 2090–2099 decade). Therefore, we showed a strong short- and medium-term response for these species, highlighting the need of short-term limitation of climate change (e.g. RCP2.6 peak-and-decline scenario). Concerning the Mediterranean Sea, we projected a north-westward distribution shift for all cephalopod species (Fig. 3) in accordance with the high ESI decrease in the southwestern basin (Fig. 3). However, this shift is less pronounced in the Mediterranean compared to Atlantic façade (i.e. + 0.30° maximum; Fig. 3). Despite the non-linear coastline (e.g. Adriatic Sea) in the Mediterranean that may influence centroid shifts, the distributional centroid shift in the Mediterranean Sea gradually increases over time and with warming intensity (Fig. 3), confirming the necessity to contain global warming under 2 °C (i.e. RCP2.6).

## Discussion

### Cephalopod: environment interactions

For all species, we highlighted an opposite response to climate change between northern and southern Europe (i.e. respectively north and south of the English Channel). We also highlighted the necessity of limiting global warming—therefore its impact on species distribution—at the lowest possible level (i.e. RCP2.6) by the end of the century. In a context of severe warming (i.e. + 4 °C in the North Sea, + 2 °C in the Bay of Biscay and + 4 °C in the Mediterranean Sea by 2100 under RCP8.5 conditions; Supplementary Appendix 5), the range of SBT that are the most suitable for these cephalopods (i.e. 10 to 13 °C)^5^ were projected to shift from the Bay of Biscay and the Celtic Sea to the Norwegian coasts. While cephalopod egg survival is stable within the thermal limits of the species (i.e. distribution centre), temperature is driving major population dynamic processes at the thermal limits of the species (i.e. projected distribution edge)^10,84^. In the Mediterranean, severe warming may induce higher metabolic rate^85,86^ at the cost of lower amounts of yolk in the eggs^18^, therefore lower embryonic survival and higher starvation risk at the para-larvae stage^10^. In northern Europe, we projected future suitable conditions at the lower thermal limit of cephalopods. Cephalopods reproducing at their lower thermal limit are characterised by larger eggs and higher embryonic survival rate^85–87^. While their slower growth and metabolic rate may induce higher predation mortality in their early lifestages, adult individuals produce more eggs, contributing to population expansion in suitable low temperature areas^10^. Moreover, regions characterised by an important temperature variability (e.g. eastern North Sea, Kattegat and the Gulf of Gabès) may be less suitable as cephalopods are highly sensitive to temperature (ectotherms), especially during their embryonic and paralarvae stages, impacting their recruitment success^5–7,10,22^.

### Methodological limitations and perspectives

In addition to the methodological improvements retrieved from Schickele et al.^45^, it appeared that for cephalopod species, a larger permeability (i.e. excluding outer quantile) of the convex hull generally improved model quality (see Supplementary Appendix 8). We assumed that for widely distributed species, a larger permeability may induce less constrained pseudo-absences (i.e. a low diversity of environmental conditions compared to the number of pseudo-absences necessary for model calibration)^73,88^, avoiding an artificial threshold type response along the distributional edge. We encourage further testing of this pseudo-absence selection hypothesis on other species (e.g. effect of the number of factors, rare species, wider range of restricted convex hulls to be tested). Despite an improved modelling framework, limitations inherent to SDMs may explain divergences between ESI values (i.e. potential distribution) and observed biomass (i.e. realised distribution) at local scale only^48^, such as habitat availability and trophic interactions. Conversely to pelagic species, the distributional range of common octopus and common cuttlefish may be locally affected by benthic parameters such as the availability of solid substrates (e.g. rocks, shells, anthropogenic litter) for their settlement and reproduction^7^. Moreover, their early lifestages (e.g. para-larvae) are particularly sensitive to prey availability^10,18^ and predation^4,89^, differentiating the realised distribution from the potential distribution. We acknowledge that these limitations may influence the realised niche at local scale^48^, a perspective constrained by oceanographic surveys, biological and habitat data large scale availability^90^. Therefore, in the context of local scale and conservation focused studies, we encourage coupling our results with habitat factors such as the availability of rocky bottom or seagrass cover that may complement our SDM projections on local scale (i.e. high resolution habitat factor for local hierarchical filtering)^70^. To better estimate the local realised distribution of cephalopods, we also encourage to include predator and prey availability in a multi-model approach^91–93^. Finally, one could argue that an ensemble modelling evaluation procedure based on independent historical data (i.e. hindcasting)^94^ is an interesting validation perspective to test the robustness of our predictions and shift our baseline by the early industrial era. However, it is limited by the large-scale availability of historical cephalopod observation data (e.g. early twentieth century) at the thermal limits of the species (i.e. truncated validation dataset). Nevertheless, in a precautionary approach, our multi-SDM, multi-GCM and multi-RCP^43^ projections provide necessary information for ecosystem managers and fisheries stakeholders to anticipate medium to long-term climate-induced change on these important species^34,95^.

### Ecological and fisheries implications

Cephalopods, including the three studied taxa, have a central role in ecosystems^2–4^, especially in the Mediterranean Sea and northern Atlantic ocean^2^. Following our future ESI projections, future distributional shifts of cephalopods may induce important modifications on food-web functioning. As suggested for similar trophic level and keystone species, the biomass of cephalopod may follow the same temporal variations as the ESI in a given geographical area. In this context, the projected northward distributional range extension may induce increasing top-down impacts on lower trophic levels (e.g. crustacean, planktivorous fish), potentially influencing the productivity of these lower trophic level species^96,97^. On the contrary, southern areas (e.g. the Mediterranean Sea) may see (i) a decrease in the top-down control on benthic communities, leading to their development coupled with (ii) a biomass decreases of cephalopods, forcing their predators to forage on other species (e.g. small pelagic fishes), with potential synergistic effects with fisheries. Indeed, cephalopods are supporting important fisheries, especially in the Mediterranean Sea and in the North Sea^28,35,98^. In the context of climate change, an increase in temperature and in the frequency of extreme climatic events may greatly influence cephalopod recruitment^10^, that is already known for its inter-annual variability^84^. The perspective of sustainable and precautious fisheries management^34,99^ has driven the development of recent stock assessment procedure including temperature-induced recruitment variability. Our future projections and centroid evolution provide valuable mid- and long-term information to complement classical stock assessment by identifying geographical areas and species that may experience (i) future variation in environmental suitability (i.e. affecting species abundance) or (ii) a distributional range shift (i.e. affecting the stock extent). Additionally, we strongly encourage further local scale and biomass-based studies such as habitat or lifecycle models in the areas we identified as largely impacted by climate change. These local scale approaches are largely complementary with SDMs, allowing a pluri-specific and operational assessment of the impacts of climate change on species, fisheries and ecosystems identified as the most sensitive to climate change. Because the socio-economy of several countries and coastal regions directly depend on the yield of their fisheries ^100,101^—that emphasises their vulnerability to climate change^30,31^—our results provide a first assessment of the local cephalopod fisheries that may be vulnerable to climate change. We provided spatially explicit projections of both contemporary and climate induced distribution throughout the twenty-first century—that we encourage to complement with habitat, ecosystem and socio-economic models—to anticipate medium- to long-term management and conservation challenges (e.g. geographical redefinition of the stocks, adaptation of fishing grounds and targets)^34,102^.

## Supplementary Information

Supplementary Information.
