# Supplementary material for: European cephalopods distribution under climate-change scenarios
Source: Sci Rep. 2021 Feb 16;11:3930. doi: 10.1038/s41598-021-83457-w (PMC7886854; doi:10.1038/s41598-021-83457-w)
Supplement: Supplementary file 1 — Supplementary Information. [file 41598_2021_83457_MOESM1_ESM.pdf]

Journal : Scientific Reports

Article type: Original research

Title: **European cephalopods distribution under climate-change scenarios**

Authors: Alexandre Schickele[1*], Patrice Francour[1] & Virginie Raybaud[1]

**Corresponding author*

[1]*Université Côte d'Azur, CNRS, UMR 7035 ECOSEAS, Nice, France.*

APPENDIX 1

The occurrence data retrieved from the GBIF database are provided hereafter:

Octopus vulgaris : GBIF.org (6/12/2018) GBIF Occurrence Download (10.15468/dl.zcqihl)

Sepia officinalis : GBIF.org (6/12/2018) GBIF Occurrence Download (10.15468/dl.amgjax)

Loligo vulgaris : GBIF.org (6/12/2018) GBIF Occurrence Download (10.15468/dl.jzhgnm)

Supplementary Table 1: Additional references used to complete each species observation datasets.

Species	Geographical area	Number occurrence considered	Reference
Common octopus *Octopus vulgaris*	Senegal	20	Domain et al. (2000)
	Ascension Island	6	Barnes (2017)
	Brazil	11	Moreira et al. (2011)
	Japan	3	Sakaguchi et al. (1999)
	South Korea	2	Kim et al. (2008)
Common cuttlefish *Sepia officinalis*	Andaman Sea	1	Muchlisin (2014)
	Mozambique channel	6	Ménard et al. (2013)
	Gabon	10	Bianchi (1992)
Common squid *Loligo vulgaris*	Gabon	10	Bianchi (1992)
	South Morocco	4	Arkhipkin (1995)
	Portugal	17	Morais da Cunha et al. (1995)
	Adriatic Sea	10	Sifner and Vrgoc (2004)
	Adriatic Sea	14	Sifner et al. (2005)
	Aegean Sea	15	Tserpes et al. (1999)
	Aegean Sea	16	Lefkaditou and Kaspiris (1998)
	North-East Levantine Sea	4	Duysak et al. (2008)

Note that for data georeferenced from publication figures, the number of occurrences considered in the models may be underestimated in comparison of the number of observed specimens. This is explained by a higher field sampling resolution compared to the resolution of our environmental filtration procedure and the spatial resolution of our environmental parameters.

References

Arkhipkin A. (1995) Age, growth and maturation of the european squid loligo vulgaris (Myopsida, loliginidae) on the west saharan shelf. J. mar. Biol. Ass. U.K., 75:593-604

Barnes, D. K. A. 2017. Marine colonization and biodiversity at Ascension Island and remote islands. - Journal of the Marine Biological Association of the United Kingdom 97: 771–782.

Bianchi, G. 1992. Demersal assemblages of the continental shelf and upper slope of Angola. - Marine Ecology Progress Series 81: 101–120.

Domain, F. et al. 2000. Growth of Octopus vulgaris from tagging in Senegalese waters. - J. Mar. Biol. Ass. 80: 699–705.

Duysak O., Sendao J., Bores T., Tureli C. and Erdem U. (2008) Cephalopod distribution in Iskenderun Bay (Eastern Mediterranean-Turkey), Journal of Fisheries Sciences, 2(2):118-125

Kim, Y. et al. 2008. Reproductive Biology of Common Octopus, Octopus vulgaris in the South Sea of Korea. - Korean J. Malacol. 24: 161–166.

Lefkaditou E. and Kaspiris P. (1998) Distribution and Reproductive Biology of Sepietta neglecta (Naef, 1916) (Cephalopoda: Sepioidea) in the North Aegean Sea (Eastern Mediterranean). The Veliger. 41(3):239-246

Ménard, F. et al. 2013. Pelagic cephalopods in the western Indian Ocean: New information from diets of top predators. - Deep Sea Research Part II: Topical Studies in Oceanography 95: 83–92.

Morais da Cunha M., Moreno A. and Pereira J. (1995) Spatial and temporal occurrences of Loligo spp in portuguese waters. ICES C.M.

Moreira, A. A. et al. 2011. Evidence for genetic differentiation of Octopus vulgaris (Mollusca, Cephalopoda) fishery populations from the southern coast of Brazil as revealed by microsatellites. - Journal of Experimental Marine Biology and Ecology 407: 34–40.

Muchlisin, Z. A. 2014. Morphometric variations of three species of harvested cephalopods found in northern sea of Aceh Province, Indonesia. - Biodiversitas, Journal of Biological Diversity 15: 142–146.

Sakaguchi, H. et al. 1999. Occurrence of planktonic juveniles of Octopus vulgaris in the eastern Iyo-Nada of the Seta inland sea, Japan. - Bulletin of the Japanese Society of Fisheries Oceanogrpahy (Japan) 63: 181–187.

Sifner S. and Vrgoc N. (2004) Population structure, maturation and reproduction of the European squid, Loligo vulgaris, in the Central Adriatic Sea. Fisheries Research. 69:239-255

Sifner, S. et al. 2005. COMPOSITION AND DISTRIBUTION OF THE CEPHALOPOD FAUNA IN THE EASTERN ADRIATIC AND EASTERN IONIAN SEA. - Israel Journal of Zoology 51: 315–330.

Tserpes, G. 1999. Species distribution in the southern Aegean sea based on bottom-trawl surveys. - Aquatic Living Resources 12: 167–175.

APPENDIX 2:

Supplementary Table 2: Evaluation metrics for all environmental factors combinations for the considered convexhull (10;90).
Black cells correspond to algorithms with a CBI<0.5. Grey cells correspond to algorithms with a CBI>0.5 but an irrelevant response curve. White cells correspond to validated algorithms considered in the ensemble model. For each species, the final ensemble model considered for future projections has its CBI indicated in bold.

		A-posteriori filtration		Algorithms								Evaluation
	**Environmental parameters**	**Bathymetry (-300 m)**	**Distance to coast (50 km)**	**GLM**	**GBM**	**GAM**	**ANN**	**RF**	**FDA**	**MARS**	**NPPEN**	**CBI**
Common octopus	SBT, SBTr	Yes	No									**0.85**
	SBT, SBTvar											0.74
	SBT, SSS											0.69
	SBT, SBTr, SSS											0.61
	SBT, SBTvar, SSS											
	SBT, SBTr		Yes									0.81
	SBT, SBTvar											0.73
	SBT, SSS											
	SBT, SBTr, SSS											0.71
	SBT, SBTvar, SSS											0.56
Common cuttlefish	SBT, SBTr	Yes	No									0.74
	SBT, SBTvar											0.79
	SBT, SSS											0.64
	SBT, SBTr, SSS											**0.89**
	SBT, SBTvar, SSS											0.84
	SBT, SBTr		Yes									0.83
	SBT, SBTvar											0.77
	SBT, SSS											0.65
	SBT, SBTr, SSS											0.87
	SBT, SBTvar, SSS											0.82
Common squid	SBT, SBTr	Yes	No									0.73
	SBT, SBTvar											0.78
	SBT, SSS											0.64
	SBT, SBTr, SSS											0.85
	SBT, SBTvar, SSS											0.71
	SBT, SBTr		Yes									0.82
	SBT, SBTvar											0.70
	SBT, SSS											0.65
	SBT, SBTr, SSS											**0.89**
	SBT, SBTvar, SSS											0.79

APPENDIX 3

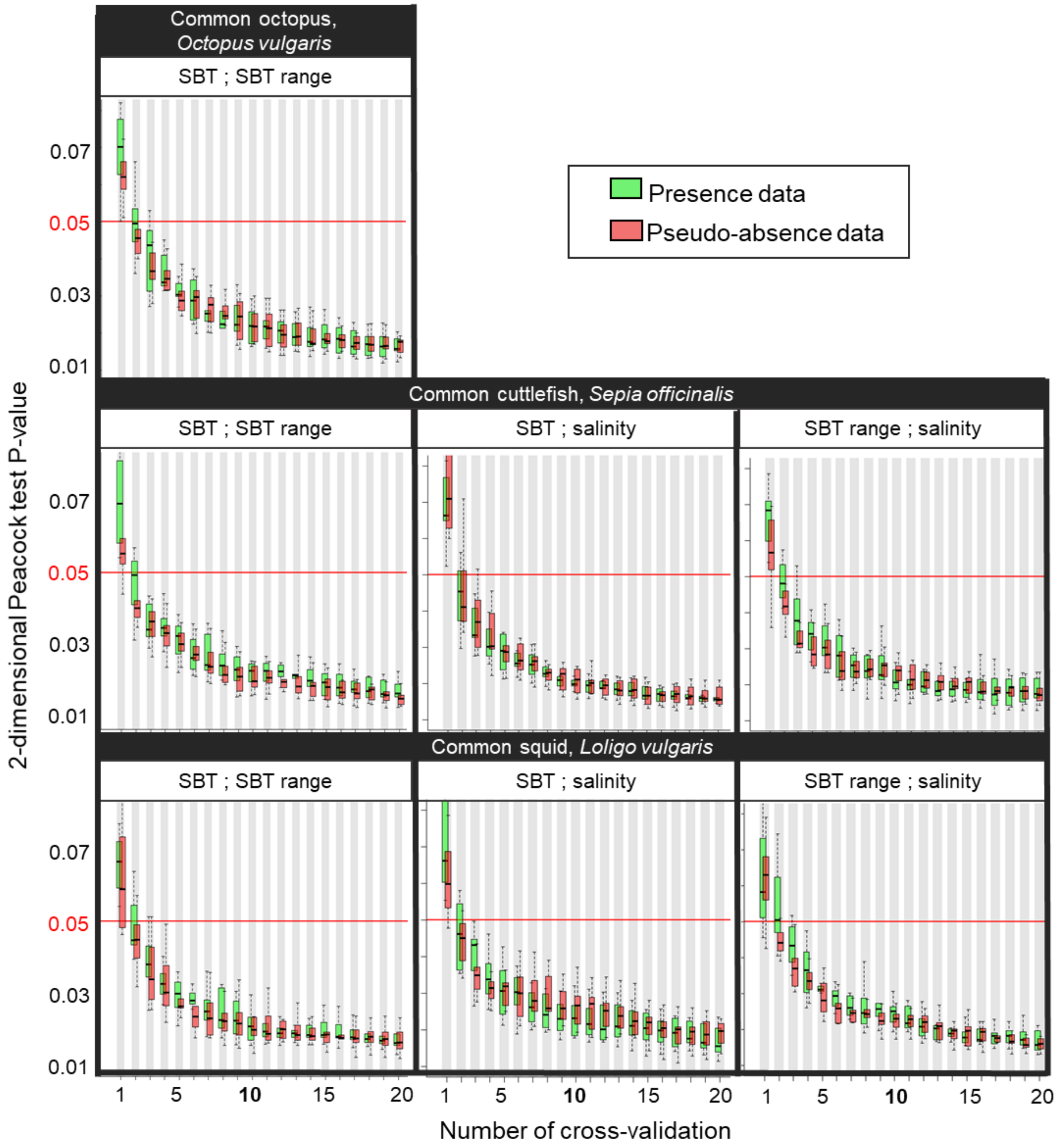

Supplementary Figure 1 : Barplots representing the result of two-dimensional Peacock test between the data retrieved from the post-filtration procedure and the calibration data retrieved from the n-number of cross-validation. The test was performed for presences and pseudo-absences separately. For each number of cross-validation runs, the procedure has been repeated 10 times. A Peacock test value under 0.05 indicates that both multi-dimensional samples follow the same common distribution.

In order to test if the calibration data retrieved from the 10 cross-validation runs is representative of the post-filtration dataset, we performed a two-dimensional Peacock test (Peacock, 1983). The latter is an adaptation of the Kolmogorov-Smirnov test (Smirnov, 1948) that evaluates if two multi-dimensional samples are drawn from the same distribution. For all datasets (i.e. presences and pseudo-absences), we showed that the calibration datasets and the post-filtration datasets followed the same two-dimensional distribution (95 % confidence threshold) for a number of cross-validation runs above 5. Moreover, we highlighted a low evolution of the p-values for higher number of cross-validation runs. We acknowledge that testing the effect of the number of cross-validation runs on the species distribution model output data may provide a more accurate estimation of the minimum number of cross-validation runs. However, performing species distribution models with higher number of cross-validation runs (e.g. 20, 50, 100) for each combination of environmental parameters to be test is of difficult applicability when considering the computational intensity.

References:

Peacock (1983) Two-dimensional goodness-of-fit testing in astronomy. *Monthly Notices of the Royal Astronomical Society*. 202(3): 615-627

Smirnov (1948) Table for Estimating the Goodness of Fit of Empirical Distributions. *Annals of Mathematical Statistics*. 19(2): 279-281.

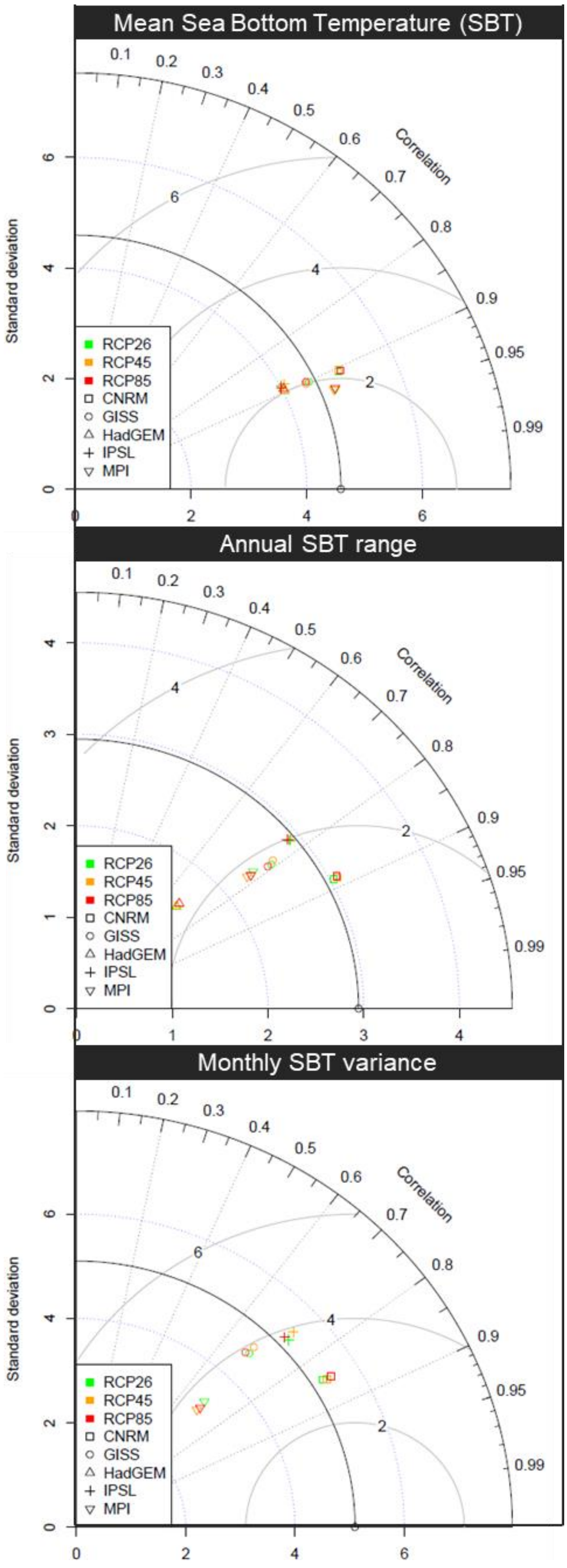

APPENDIX 4

Supplementary Figure 2: Taylor diagram corresponding to the uncorrected Sea Bottom Temperature (SBT) data. With a the mean SBT, b the annual SBT range and c the monthly SBT variance. Taylor diagrams were generated by A.S. using the R v3.4.4 software (R Core Team, 2018; https://www.R-project.org/), specifically the "raster" and "plotrix" package.

The diagrams present the General Circulation Model (GCM) based data before our bias reduction procedure (i.e. green, orange and red pictograms; see **Table 1** for references). Because of heterogenous standard deviation and a high mean square root difference (RMSD; >2°C) our environmental suitability projections may have been biased in several geographical areas. Therefore, we corrected the GCM-based data in order to match the contemporary data (i.e. black circle at the bottom of each diagram), defined by a standard deviation equal to the contemporary data, a RMSD of 0 and a correlation of 1.

APPENDIX 5

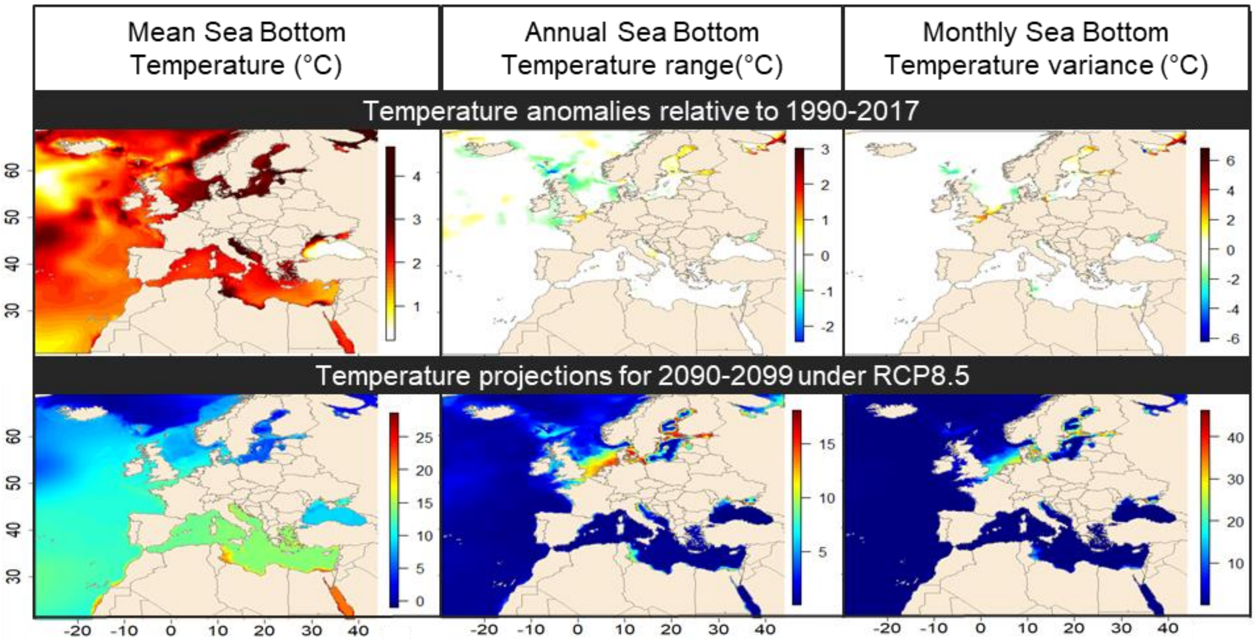

Supplementary Figure 3: Temperature related parameters evolution (°C) between 1990-2017 and 2090-2099 under RCP8.5. The top panels (**a**) correspond to the calculated temperature anomaly relative to 1990-2017. The bottom panels (**b**) correspond to late century temperature projections for 2090-2099 under RCP8.5; note the different scale in each plot. Maps were generated by A.S. using the R v3.4.4 software (R Core Team, 2018; https://www.R-project.org/), specifically the “raster” and “maptools” package. World borders were retrieved from http://thematicmapping.org.

APPENDIX 6

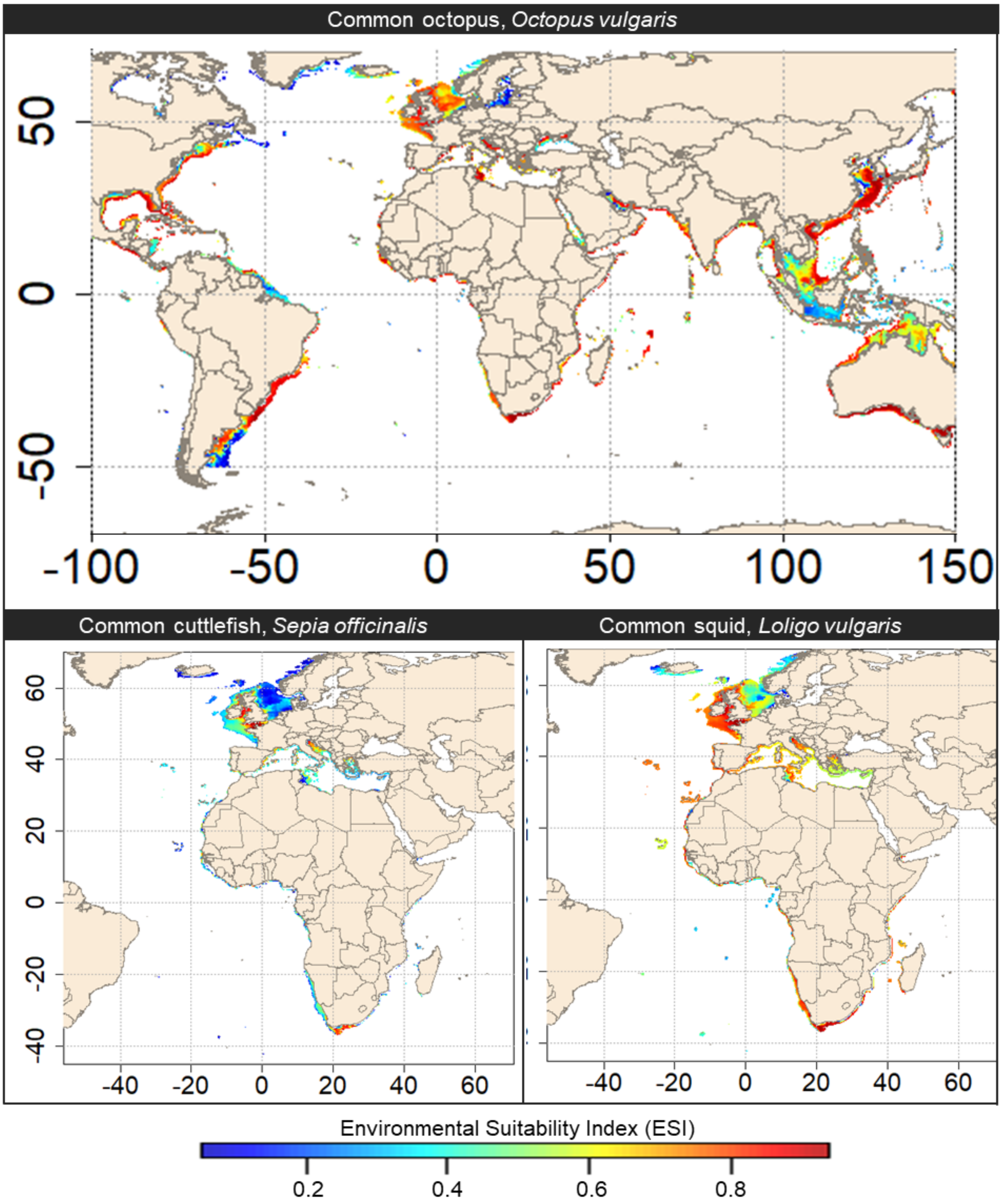

Supplementary Figure 4: Contemporary (1990-2017) environmental suitability index on the entire distribution range of common octopus, common cuttlefish and common squid. Note that a narrow distance to coast threshold has been added for common octopus and common cuttlefish for visual purposes only because of the coarse (i.e. 0.1°) coastal resolution. Maps were generated by A.S. using the R v3.4.4 software (R Core Team, 2018; https://www.R-project.org/), specifically the "raster" and "maptools" package. World borders were retrieved from http://thematicmapping.org.

APPENDIX 7

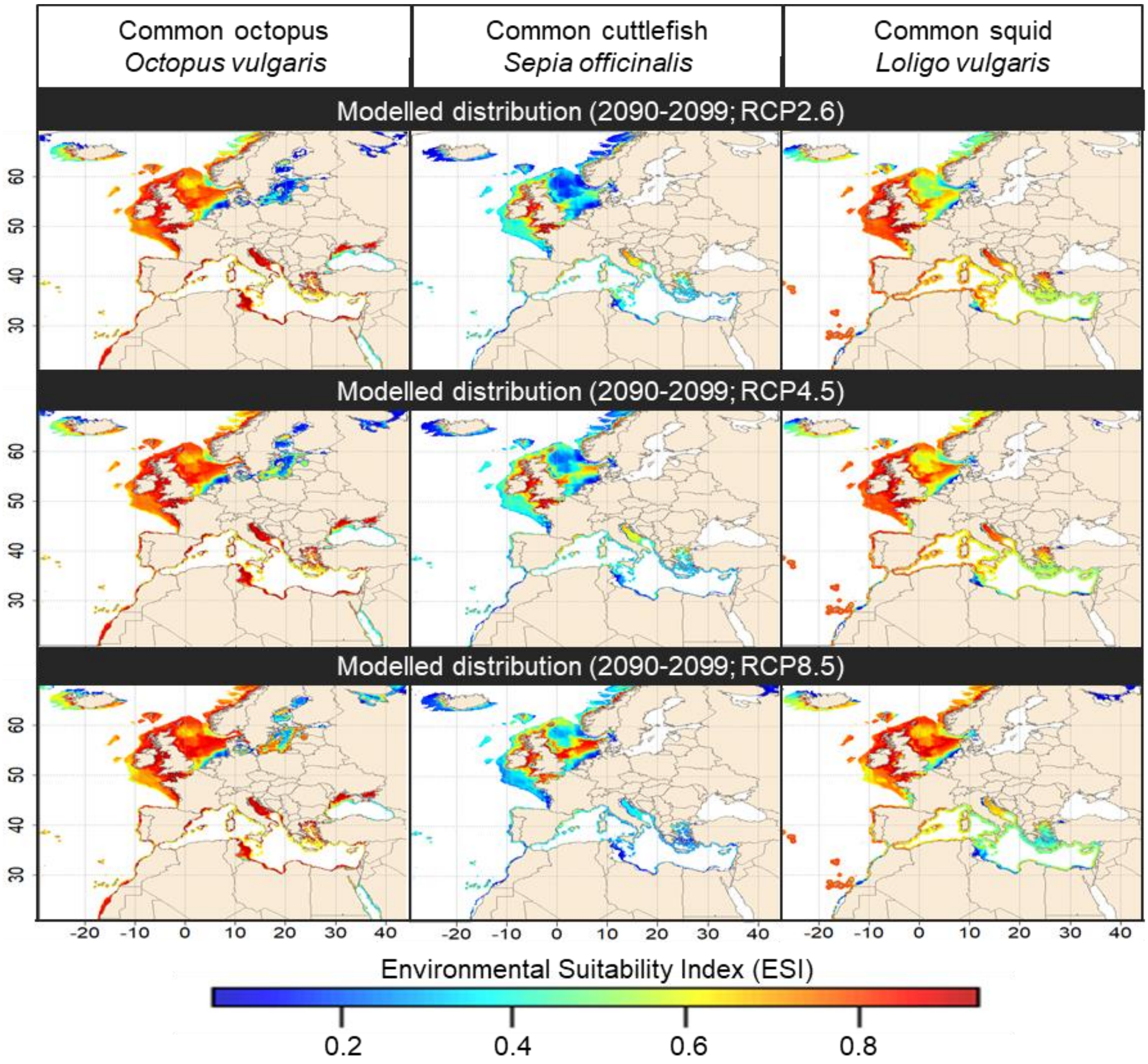

Supplementary Figure 5: Future (2090-2099) environmental suitability projections for Europe, under RCP2.6, 4.5 and 8.5 conditions. Note that a narrow distance to coast threshold has been added for common octopus and common cuttlefish for visual purposes only because of the coarse (i.e. 0.1°) coastal resolution. Maps were generated by A.S. using the R v3.4.4 software (R Core Team, 2018; https://www.R-project.org/), specifically the “raster” and “maptools” package. World borders were retrieved from http://thematicmapping.org.

APPENDIX 8

Supplementary Table 3: effect of the convex hull quantiles on Continuous Boyce Index. With SBT (Sea Bottom Temperature) and SSS (Sea Surface Salinity).

	**Common octopus** *Octopus vulgaris*	**Common cuttlefish** *Sepia officinalis*	**Common squid** *Loligo vulgaris*
**Environmental parameters**	SBT + SBT range	SBT + SBT range + SSS	SBT + SBT range + SSS
**Restricted convex hull (2.5; 97.5)**	0.73	0.91	0.80
**Restricted convex hull (5; 95)**	0.81	0.89	0.84
**Restricted convex hull (10; 90)**	0.85	0.89	0.85
